# Metagenomic changes in response to antibiotic treatment in severe orthopedic trauma patients

**DOI:** 10.1016/j.isci.2024.110783

**Published:** 2024-08-22

**Authors:** Afroditi Kouraki, Amy S. Zheng, Suzanne Miller, Anthony Kelly, Waheed Ashraf, Davide Bazzani, Angela Bonadiman, Guendalina Tonidandel, Mattia Bolzan, Amrita Vijay, Jessica Nightingale, Cristina Menni, Benjamin J. Ollivere, Ana M. Valdes

**Affiliations:** 1 Academic Unit of Injury, Recovery and Inflammation Sciences, School of Medicine, University of Nottingham, Nottingham NG7 2UH, UK; 2 NIHR Nottingham Biomedical Research Centre, Nottingham University Hospitals NHS Trust and the University of Nottingham, Nottingham NG7 2UH, UK; 3 Prebiomics srl, 38123 Trento, Italy; 4 Department of Twin Research, King’s College London, London SE1 7EH, UK

**Keywords:** Trauma, Genomics, Microbiome

## Abstract

We investigated changes in microbiome composition and abundance of antimicrobial resistance (AMR) genes post-antibiotic treatment in severe trauma patients. Shotgun sequencing revealed beta diversity (Bray-Curtis) differences between 16 hospitalized multiple rib fractures patients and 10 age- and sex-matched controls (*p* = 0.043), and between antibiotic-treated and untreated patients (*p* = 0.015). Antibiotic-treated patients had lower alpha diversity (Shannon) at discharge (*p* = 0.003) and 12-week post-discharge (*p* = 0.007). At 12 weeks, they also exhibited a 5.50-fold (95% confidence interval [CI]: 2.86–8.15) increase in *Escherichia coli* (*p* = 0.0004) compared to controls. Differential analysis identified nine AMRs that increased in antibiotic-treated compared to untreated patients between hospital discharge and 6 and 12 weeks follow-up (false discovery rate [FDR] < 0.20). Two aminoglycoside genes and a beta-lactamase gene were directly related to antibiotics administered, while five were unrelated. In trauma patients, lower alpha diversity, higher abundance of pathobionts, and increases in AMRs persisted for 12 weeks post-discharge, suggesting prolonged microbiome disruption. Probiotic or symbiotic therapies may offer future treatment avenues.

## Highlights

• Lower diversity in antibiotic-treated versus untreated trauma patients and controls
• Antibiotic-treated patients had higher abundance of dance of pathobiontsgens like *E.coli*
• Antibiotic-treated patients had higher abundance of antimicrobial resistance genes
• These changes persisted at 12 weeks post-discharge suggesting microbiome disruption

## Graphical abstract

**Figure:**
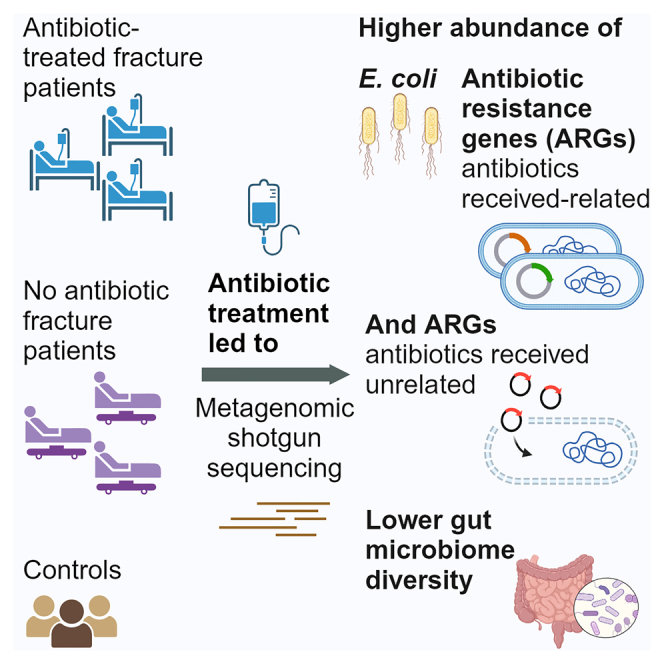

## Introduction

Postsurgical infection is one of the major complications after bone fracture fixation surgery. Post-traumatic infection is a devastating complication associated with significant morbidity, mortality, and healthcare costs.^1^^,^^2^^,^^3^ Mortality rates can reach up to 50% in vulnerable groups,^4^ and, despite advancements in asepsis, surgical site infection rates remain elevated, ranging from 1% to 2%^5^ in lower-risk fractures to 23% in higher-risk open fractures,^6^^,^^7^^,^^8^^,^^9^ surpassing infection rates in other surgeries such as hip procedures.^10^

National guidance recommends prophylactic antibiotics and infection control for all implant surgeries,^11^^,^^12^ yet infection remains a major risk, while antibiotic use poses additional risks such as *C. difficile* infection and antimicrobial resistance (AMR), recognized globally by the World Health Organization.

The human gut harbors a diverse ecosystem of microorganisms, collectively known as the gut microbiome, which play essential roles in metabolism, signaling, and immune regulation.^13^ Both commensal and pathogenic components of the gut microbiome act as repositories for AMR genes, posing risks to host health and facilitating their spread to other microbes and the environment. AMR is a growing concern globally,^14^ with antibiotic-induced changes to the gut microbiome linked to various gastrointestinal diseases.^15^ Additionally, in critically ill patients, disruptions to the gut microbiome can contribute to sepsis by allowing bacteria and their byproducts to cross the intestinal barrier.^16^ Moreover, there is an important role of the gut microbiome in the development of osteoporosis, which is a significant risk factor for fractures.^17^^,^^18^^,^^19^

Pilot studies testing the effects of antibiotics on gut microbiome composition and on AMR in healthy adults have already been reported. For instance, five-day administration of 4 different antibiotic regimens resulted in an acute decrease of microbial richness in the gut which was fully restored within 60 days.^20^ Gut microbiome diversity on admission in severely injured patients is predictive of a variety of clinically important outcomes including mortality, length of stay, and need for ventilation.^21^ Injured patients develop changes in gut microbiome composition within 72 h of trauma, characterized by depletion of certain bacterial orders (*Bacteroidales*, *Fusobacteriales*, and *Verrucomicrobiales*) and significant increases in *Clostridiales* and *Enterococcus*.^22^ There is a need for a comprehensive assessment within the context of surgical patients who have undergone antibiotic treatment.

Although metagenomics data following antibiotic use in orthopedic and trauma surgery are limited, European surveillance data indicate that 39% of infections, including surgical site infections, are caused by carbapenem-resistant bacteria, including carbapenem-resistant *Enterobacteriaceae*.^23^ The class *Gammaproteobacteria* contains numerous gram-negative human pathogens, especially the family *Enterobacteriaceae*, which are prominent pathogens in postsurgical infections like diarrhea, septicemia, and pneumonia.^16^^,^^24^

Importantly, the gut microbiome can help prevent the colonization of the gut by pathogens,^25^ and a highly diverse microbiota makes the gut more resistant to colonization by pathogens that may cause infection in the gut or after translocation elsewhere in the body.^26^

Given the role of the gut microbiome in modulating AMR and at the same time providing a pool of potential pathobionts that can contribute to postsurgical infections, it is important to understand the effect of antibiotic treatment on the gut microbiome in surgical patients, the medium-term effect post hospital discharge, not just in terms of microbial diversity but also in terms of abundance of the most common pathogenic species and AMR genes.

In this pilot study, we analyzed data from an ongoing randomized controlled trial (RCT) investigating whether surgical repair of multiple rib fractures after severe chest injury reduces mortality.^27^ These fractures often accompany chest wall injuries, leading to respiratory impairment, commonly referred to as “flail chest.”^28^^,^^29^ Traditionally, patients with a flail chest receive conservative treatment, i.e., pain management, physiotherapy, and, if necessary, assisted ventilation, along with daily multidisciplinary assessment.^30^ More recent orthopedic guidance suggests also considering surgical stabilization along with conservative measures for flail chests.^31^^,^^32^

While prophylactic antibiotics are standard in surgical procedures, patients with a flail chest do not always receive surgical treatment and hence do not necessarily receive antibiotics unless an infection develops. Using pilot microbiome metagenomics data from the ongoing RCT,^33^ we were able to compare severe trauma patients (flail chest) who received antibiotics during their hospital stay with those who did not, alongside age- and sex-matched controls from two separate studies.

The aim of this study was therefore to assess the effect of antibiotic use on gut microbiome diversity and composition as well as the abundance of AMR genes in fracture patients with similar levels of trauma upon admission, comparing those who received antibiotics with those who did not, alongside control subjects. Our study may provide valuable insights into the interplay between antibiotic usage, gut microbiome dynamics, and AMR acquisition in the context of severe orthopedic trauma.

## Results

**Figure 1:**
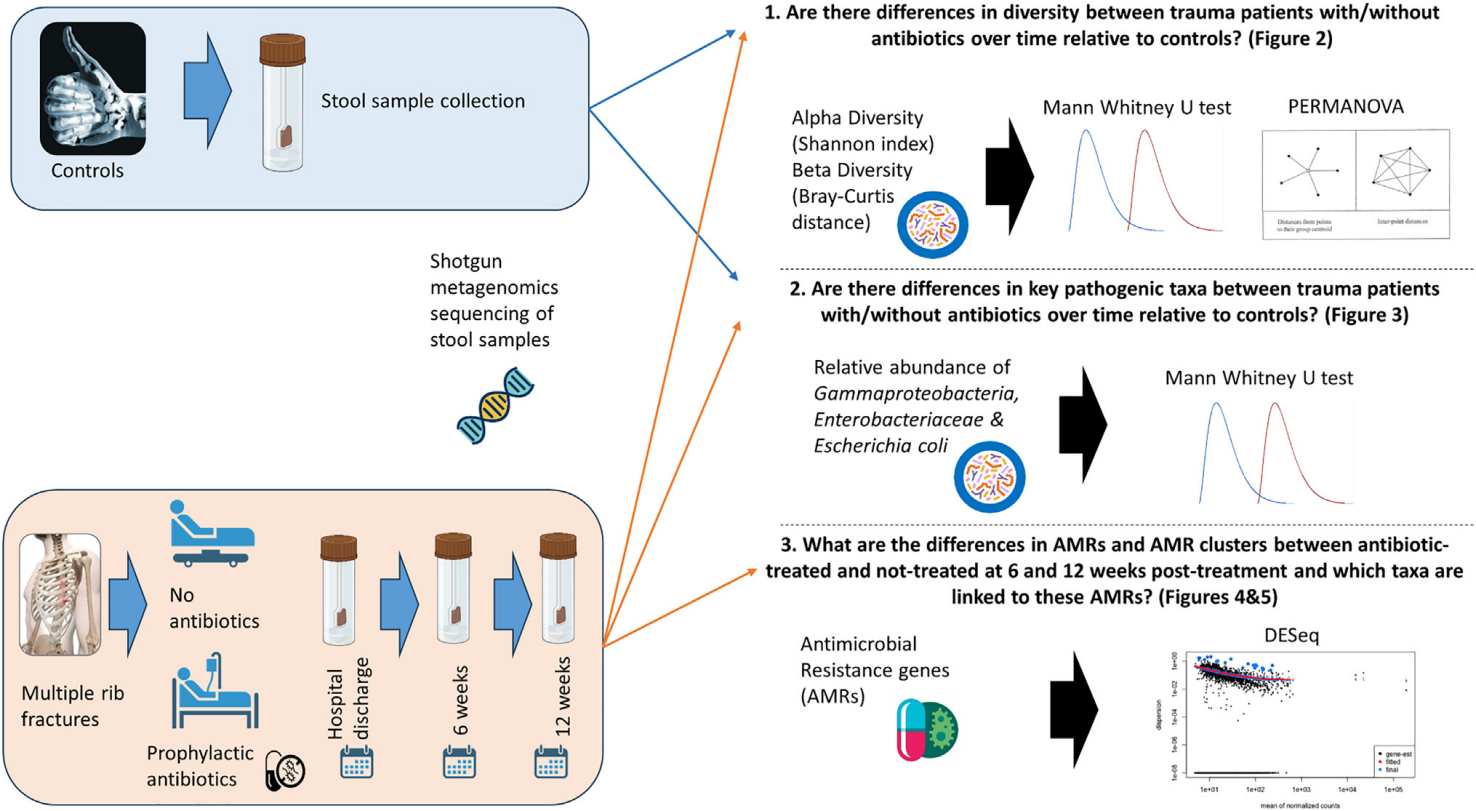
Flow chart of the study design

A flowchart of the study design is presented in Figure 1. The descriptive characteristics of the rib fracture patients and controls, and the use of antibiotics, are included in Table S1. Controls were matched to the antibiotic group in terms of age (64 years on average) and sex (50% male). All individuals in the antibiotic group received intravenous (IV) flucloxacillin for 24 h, 91% received in addition 1 dose of gentamycin, 27% also received 1 dose of Tazocin, and one individual received 5 days of co-amoxiclav (a beta-lactam antibiotic and clavulanic acid, a beta-lactamase inhibitor). Flucloxacillin is a beta-lactam antibiotic^34^; gentamycin is an aminoglycoside antibiotic^35^; Tazocin is a combination of piperacillin, a beta-lactam antibiotic, and tazobactam,^36^ a beta-lactamase inhibitor; and co-amoxiclav is the combination of a beta-lactam antibiotic and clavulanic acid, a beta-lactamase inhibitor.^37^

### Differences in measures of diversity

**Figure 2:**
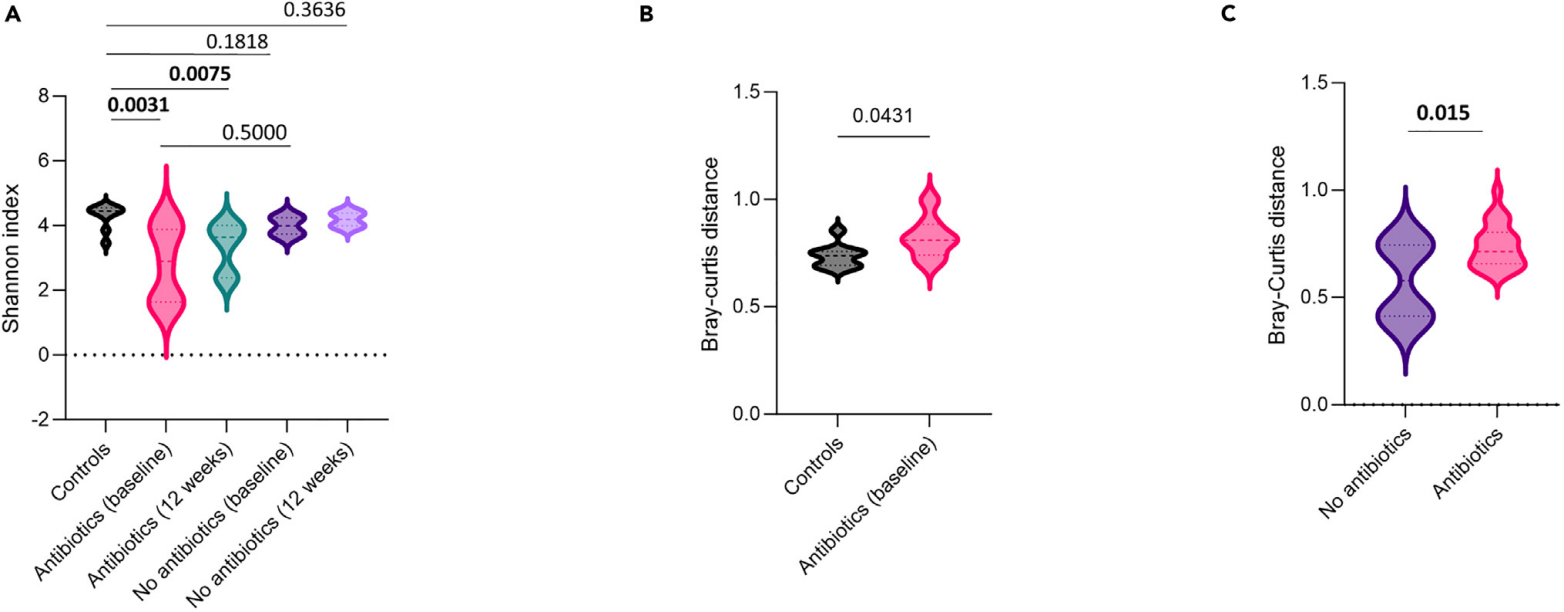
Violin plots showing differences in alpha and beta diversity within groups (A–C) (A) Differences in alpha diversity (Shannon’s index) between healthy controls, surgical patients who received prophylactic antibiotics, and surgical patients who did not receive prophylactic antibiotics at baseline and 12 weeks. Statistical significance was assessed using pairwise comparisons between controls and treated and untreated patients both at baseline and 12 weeks, as well as between treated and untreated patients at baseline with Mann-Whitney U tests (*p* values from Mann-Whitney U test). (B) Differences in beta diversity (Bray-Curtis distance) between the antibiotics group at the time of hospital discharge and the control group. Statistical significance was assessed using a pairwise comparison between controls and treated patients at baseline with the Mann-Whitney U test (*p* value from Mann-Whitney U test). (C) Differences in beta diversity (Bray-Curtis distance) between patients who received and did not receive antibiotic over all time points. The analysis adjusts for the time of sampling to account for temporal changes in microbial communities. The statistical significance of the differences was evaluated using PERMANOVA (permutational multivariate analysis of variance) (*p* value from PERMANOVA adjusted for time). *p* < 0.05 was considered statistically significant.

We first tested for differences in alpha and beta diversity between controls and fracture cases with and without antibiotics at the time of discharge and 12 weeks post-discharge. A significantly lower alpha diversity among fracture patients was identified in the antibiotic group at baseline and 12 weeks compared to controls, but no such difference was seen for patients who did not receive antibiotics (Figure 2A). This indicated that changes in the gut microbiome persisted over time even at 12 weeks after hospital discharge. Similarly, the Bray-Curtis index for surgical patients who received prophylactic antibiotics was significantly different from that of controls and surgical patients who did not receive antibiotics (Figures 2B and 2C). We found a significantly larger Bray-Curtis distance between surgical patients who received antibiotics at the time of discharge compared to controls, indicating a change in the microbiome occurred in the antibiotics group compared to controls. We observe a similar significant increase in Bray-Curtis between surgical patients who received antibiotics compared to ones that did not, having adjusted for the influence of time, again indicating a significant change in their gut microbiome across time points.

### Differences in Enterobacteriaceae and related taxa

**Figure 3:**
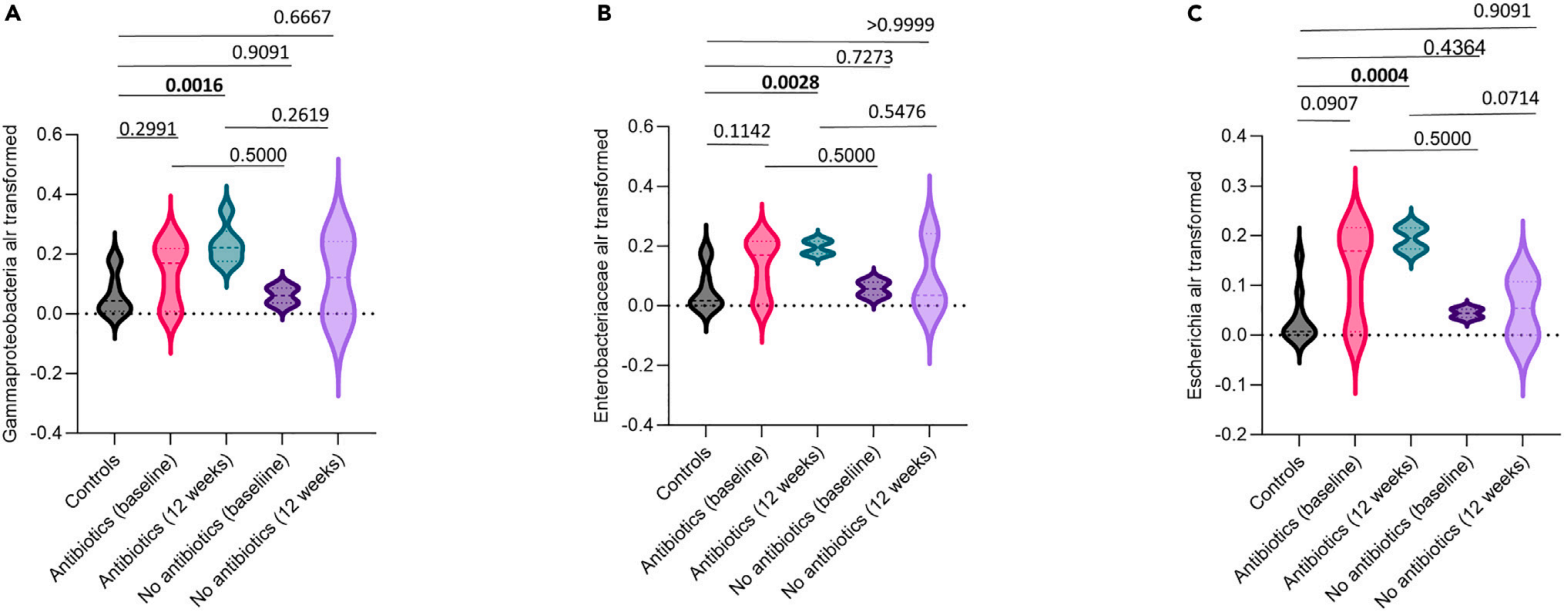
Violin plots showing differences in relative abundance of specific bacterial taxa among study groups (A–C) (A) Differences in the relative abundance (additive log transformed) of the class *Gammaproteobacteria*, (B) differences in the relative abundance (additive log transformed) of family *Enterobacteriaceae*, and (C) differences in the relative abundance (additive log transformed) of species *Escherichia coli* among healthy controls, surgical patients who received prophylactic antibiotics, and surgical patients who did not receive prophylactic antibiotics at discharge and 12 weeks post-discharge. Statistical significance was assessed using pairwise comparisons between controls and treated and untreated patients, as well as between treated and untreated patients both at baseline and 12 weeks with Mann-Whitney U tests (*p* values from Mann-Whitney U test). *p* < 0.05 was considered statistically significant.

We then tested for differences in the abundance of a specific pathogenic taxon, namely *Gammaproteobacteria*, which has been implicated in most types of postsurgical infections between controls and fracture cases with and without antibiotics at baseline and 12 weeks. At 12 weeks post-discharge, we find that compared to controls there was a significant increase in this class (Figure 3A), in the family *Enterobacteriaceae* belonging to this class (Figure 3B), and in the species *Escherichia coli*, the most abundant species in the surgical cohort, also belonging to this class (Figure 3C). This suggests that this taxon is driving at least some of the diversity changes observed in this surgical group of patients administered with antibiotics. In terms of fold change, the differences observed at baseline correspond to a 3.54-fold increase (95% confidence interval [CI] 0.89–6.18) compared to controls and 2.32 (95% CI 0.03–4.95) compared to the group who did not receive antibiotics. At 12 weeks the fold increase for the relative abundance of *E. coli* was 5.50-fold increase (95% CI 2.86–8.15) relative to controls and 3.61 (95% CI 0.98–6.24) compared with the group with no antibiotics.

### AMR genes

**Figure 4:**
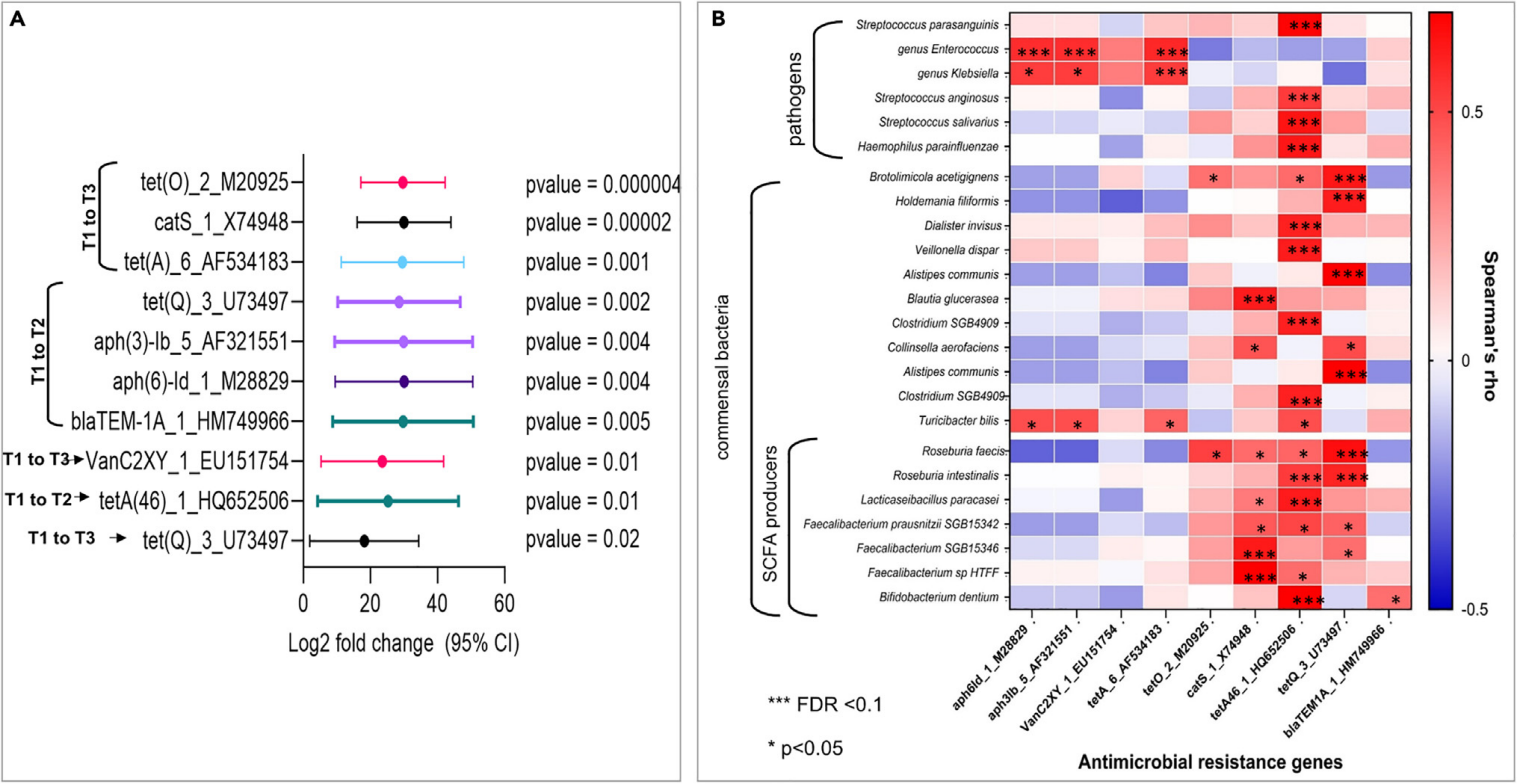
AMR genes that increased following antibiotic treatment at 6 or 12 weeks and relationships with the gut microbiome (A and B) (A) Fold changes with 95% confidence intervals (CIs) and *p* values of antimicrobial resistance genes (AMRs) that significantly changed (Wald FDR *p* values < 0.2) as a result of antibiotic use in fracture patients compared to the no antibiotic group from baseline onwards. Results from the Wald tests were used to explore two questions: “what is the difference in AMRs between antibiotics-treated and untreated at 6 weeks post-treatment?” and “what is the difference in AMRs between antibiotics-treated and untreated at 12 weeks post-treatment?”. This analysis controls for differences between groups at baseline, including random differences between the individuals from each group, which are observable before the antibiotic takes effect. The log2 fold change indicates the direction of the change in gene expression with a positive log2 fold change indicating that the expression of the AMR gene is higher in the group treated with antibiotics compared to the non-treated group at that specific time point, relative to baseline (T1). The data were normalized using the “poscounts” estimator. (B) Correlations (Spearman’s rho) between the AMRs from (A) and bacterial abundances of both commensal (including short-chain fatty acid [SCFA] producing) and pathogenic bacteria from a rarefied list of 285 species and genera (*p* values from Spearman’s rho test), ∗*p* < 0.05, ∗∗∗FDR <0.1, Scale bar ranges from −0.50 to 0.67.

Next, we investigated changes in the abundance of AMR genes over time in fracture patients who did not receive prophylactic antibiotics compared to individuals who had received antibiotics. Using differential expression we identified 9 AMR genes significantly (7 genes) or nominally significantly (2 genes) increased in the antibiotic group compared to patients who did not take antibiotics and whose abundance increased after hospital discharge at 6 or 12 weeks (Figure 4A).

### Relationships between AMR genes and the gut microbiome

We explored correlations of these AMR genes with a rarefied list of 285 species and genera and identified several significant associations (Figure 4B). We find a number of the AMRs that changed overtime in the antibiotic group showed strong positive correlations with both commensal and pathogenic bacteria. Examples of pathogenic bacteria include species of the genera *Streptococcus* and *Klebsiella* and the *Enterococcus* genus, whereas species of the genera *Faecalibacterium* and *Roseburia* and the species *Bifidobacterium dentium* are normally seen as beneficial, in particular short-chain fatty acid producers. For example, *tet(O)_2_M20925*, *catS_1_X74948*, *tetA(46)_1_HQ652506*, and *tet(Q)_3_U73497* were significantly correlated with a higher abundance of *Roseburia faecis*, whereas *aph(6)-Id_1_M28829*, *aph(3)-Ib_5_AF321551*, and *tet(A)_6_AF534183* were correlated with genera *Enterococcus* and *Klebsiella*.

### AMR gene clusters exhibiting particular patterns across groups and contribution of bacteria to their differentiation

**Figure 5:**
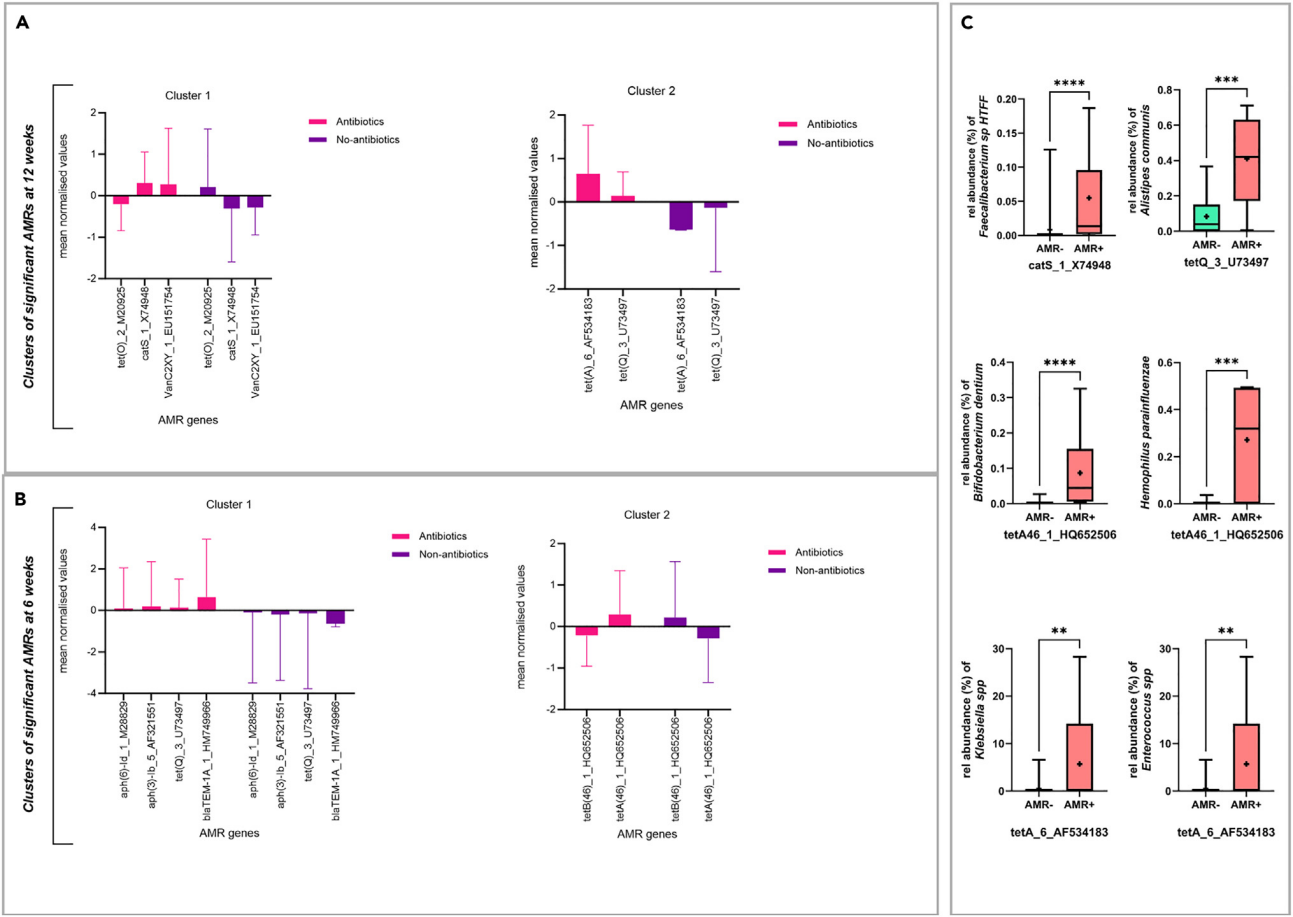
Clusters of the AMR genes with similar expression patterns across the antibiotics and non-antibiotics groups (A–C) Patterns of AMR genes clusters that exhibited significant changes in abundance between the antibiotics and non-antibiotics groups from (A) baseline to 12 weeks and (B) baseline to 6 weeks as identified through DESeq Wald tests. This analysis was used to capture changes across all time points and provide insights about genes with similar patterns of expression. Data are represented as mean normalized values +/− SD. (C) Comparisons of the percentage contribution of the relative abundance of selected pathogenic and short-chain fatty acid-producing species between AMR-negative and AMR-positive individuals of selected significantly differentiating AMRs (*p* values from Mann-Whitney U test). Data are represented as median values +/− range. ∗∗*p* < 0.01, ∗∗∗*p* < 0.001, ∗∗∗∗*p* < 0.0001.

We next used a clustering approach to identify the AMR genes significantly changed between the antibiotics and non-antibiotics groups that are associated with particular patterns across these groups based on their changes in abundance from baseline to 12 weeks (Figure 5A) and from baseline to 6 weeks (Figure 5B). DESeq was used to explore how the expression of AMR genes changed in the treated group at specific time points relative to the untreated group. “degPatterns” was used to capture changes across all time points and provide insights about genes with similar patterns of expression. For most of these genes we observe an increase in expression in the antibiotics groups but a decrease in the non-antibiotics group, apart from the genes *tet(O)_2_M20925* and *tetB(46)_1_HQ652506* which showed the opposite trend. For *tetB(46)_1_HQ652506*, this is consistent with DESeq analysis, which found a decrease in expression in the antibiotics group at both time points relative to baseline (log2 fold change T2 = −26.33 [95% CI: −47.31, −5.35] and log2 fold change T3 = −20.86 [95% CI: −38.94, −2.78]). The situation for *tet(O)_2_M20925* is more complex. Despite being clustered with genes showing an increasing trend, this gene exhibited an overall decrease in expression when considering all time points. Examining individual time point values from ‘degPatterns” reveals that the gene’s behavior deviates at specific points, showing an increase in the antibiotics group at T3 while exhibiting a decrease in expression at T1 and T2. This is consistent with DESeq results, which found a significant increase for this gene only at T3 relative to baseline (Figure 4A). The most profound overexpression was with the tetracycline-resistance gene *tet(A)_6_AF534183* which was only correlated with pathogenic genera (Figure 4B). Furthermore, abundances of pathogenic genera *Klebsiella* and *Enterococcus* also contributed to the differentiation between *tet(A)_6_AF534183*-negative and *tet(A)_6_AF534183*-positive individuals (Figure 5C). The two “*aph”* genes conferring resistance to aminoglycoside antibiotics increased in the antibiotics group at 6 weeks and were clustered together with the beta-lactamase gene, *blaTEM-1*, also increased at 6 weeks, and the tetracycline-resistant conferring gene, *tet(Q)_3_U73497*, which was increased at 6 and 12 weeks. The two aminoglycoside-conferring genes were correlated with pathogenic genera *Klebsiella* and *Enterococcus*, whereas *tet(Q)_3_U73497* was correlated with several commensal species. For example, *Alistipes*, a commensal species, increased significantly in *tet(Q)_3_U73497*-positive compared to *tet(Q)_3_U73497*-negative individuals (Figure 5C). Individuals with higher abundance of *tetA(46)_1_HQ652506* (which was clustered with *tetB(46)_1_HQ652506* but increased in the antibiotics group) showed an increased abundance of both beneficial species, such as *Bifidobacterium dentium*, and pathogenic species, such as *Haemophilus parainfluenzae*. The cephalosporins-resistance gene, *catS_1_X74948*, despite being increased in the antibiotics group, was only significantly correlated with commensal species, such as *Faecalibacterium species HTFF*.

### Link between the shift in microbiome diversity, composition, and AMRs with health endpoints

We also investigated whether there is a relationship between the shift in microbiome diversity, composition, and AMRs with demographics and health endpoints (Figure S1). We find that the Shannon index showed a positive correlation with quality of life measured with the EuroQol-5-dimension questionnaire (*p* = 0.022), which includes one question for each of the five dimensions that include mobility, self-care, usual activities, pain/discomfort, and anxiety/depression,^38^ and a negative correlation with pain (measured with a visual analog scale) (*p* = 0.0016) at the 3-month (90-day) follow-up. Conversely, *E. coli* and AMRs did not exhibit significant correlations with quality of life, pain, intensive care unit (ICU) stay, and length of hospital stay. Regarding ICU stay, only two participants required time in the ICU, one for 1 day and the other for 3 days. The average length of hospital stay was 12.16 days.

## Discussion

In this pilot study, we tested the changes in gut microbiome composition, including changes in AMR gene abundances between antibiotic-treated and non-treated individuals following severe orthopedic trauma and hospital admission for multiple rib fractures. We find that, unlike healthy individuals who receive antibiotics,^20^ the Shannon alpha diversity remains significantly lower than that in controls even 12 weeks after hospital discharge. As expected, Bray-curtis beta diversity is different between controls and antibiotic-treated groups at baseline as well as between antibiotics-treated and non-treated groups. Furthermore, we report a significantly different microbiome composition, specifically a higher abundance of class *Gammaproteobacteria* and related taxa, in the antibiotic-treated group compared to controls, as has already been reported in RCTs in infants.^39^ We find clusters of AMR genes that changed in the antibiotics group compared to the non-antibiotics group for up to 12 weeks post-discharge including genes conferring resistance to antibiotics administered for prophylaxis to the rib fracture patients with some of these AMR genes being correlated with selected commensal and pathogenic taxa.

The administration of antibiotics compromises the microbiome composition.^40^ This includes an increased abundance of potentially detrimental microorganisms, a decrease in “colonization resistance” (protection against colonization with potentially pathogenic *Enterobacteriaceae*) organisms, and the development of AMR. In addition, high antibiotic usage, while vital in managing hospital infections, can promote AMR and dysbiosis. In our study, flucloxacillin, gentamicin, Tazocin, and co-amoxiclav antibiotics were administered to the surgical fixation patients. Flucloxacillin was given to all the patients and is effective against gram-positive bacteria, including beta-lactamase producers like *Staphylococcus aureus*,^34^ and in treating bloodstream infections, endocarditis, soft tissue infections, osteomyelitis, and pneumonia.^41^ Tazocin was given to 27% of patients and combines piperacillin and tazobactam to treat a broad range of infections, including those caused by key nosocomial pathogens like *Pseudomonas aeruginosa*.^36^ Co-Amoxiclav, combining amoxicillin and clavulanic acid, treats respiratory, urinary, and skin infections^37^ and was administered to only one participant for five days. Gentamicin, administered to most patients, is used in short-term empirical combination therapy^35^ and targets gram-negative bacteria, such as *Escherichia coli*, and some gram-positive bacteria. Despite its use, *E. coli* abundance increased in treated patients. Expansion of *E. coli* following gentamycin administration has been previously documented in severely malnourished diarrhea patients^42^ and newborns treated for suspected sepsis.^43^ Overall, we report that the abundance of *Gammaproteobacteria*, specifically of *Enterobacteriaceae*, increased over time in this patient population, and this increase was driven by increased abundance of the species *Eschericha coli*, which is a common pathobiont often involved in nosocomial and post-surgical infections.^44^^,^^45^

Increases in *Enterobacteriaceae* in response to antibiotic treatment have previously been reported in neonatal infants.^39^ The species involved in the study of neonates were *Klebsiella* and *Enterococcus*, and not *Escherichia* as in our data. Interestingly, however, in our data we find that both *Klebsiella* and *Enterococcus* are positively correlated with three of the AMR genes whose abundance increased over time, consistent with observations that *Enterobacteriaceae* are extremely prone to acquire AMRs.^46^^,^^47^ These findings suggest that antibiotic treatment favors higher abundances of *Enterobacteriaceae* in the gut and that this is potentially mediated by acquisition of AMR genes.^48^^,^^49^

A previous study investigating the presence of AMRs within a hospital setting in patients receiving antibiotics used 16S rRNA sequencing alone or combined with metatranscriptomics,^50^ while other studies in critically ill patients did not specifically examine AMR dynamics post-antibiotic administration.^51^^,^^52^ Buelow et al.^50^ observed an increase of *E.coli* post-discharge on two out of the five patients who were followed up after cessation of intensive antibiotic treatment but remained in a medium-care ward and continued being treated with antibiotics. In line with this, we find an overall increase in *E. coli* following cessation of antibiotics even at 12 weeks post-discharge compared to controls, which may suggest a rapid growth of this gram-negative species that occurs upon cessation of prophylactic antibiotic therapy. Consistent with our results, they also reported an increase in *tetQ* in ICU patients. In our data, as participants were followed up at 6 and 12 weeks post-discharge, we find that this gene remains elevated even at the 12 weeks mark despite not being false discovery rate (FDR)-statistically significantly different from the group not receiving antibiotics at 12 weeks. None of the other genes Buelow et al.^50^ identified were detected in our data likely due to methodological differences; our study used a metagenomic approach whereas they used 16S and qPCR.

An intervention study focused on AMRs changes following administration of cephalosporin using a metagenomic approach in 18 healthy individuals and followed up participants for 90 days after cessation of the antibiotic.^53^ The authors reported a drastic increase in beta-lactamases, including up to a 90-fold increase of *blaTEM-1* at the end of the treatment but not at 90 days, consistent with the increased abundance of *blaTEM-1* we observed in our data at 6 but not at 12 weeks. Beta-lactamases hydrolyze the beta-lactam ring of beta-lactam antibiotics such as flucloxacillin, co-amoxiclav, and Tazocin. Given that some of these antibiotics contain beta-lactamase inhibitors, perhaps it is not surprising that no more *bla* genes were increased in our study.

In a survey of 250 *E coli* isolates from hospital patients,^54^ *blaTEM-1* was found positioned amid IS26 mobile elements, consistent with its prior identification on an *E. coli* plasmid linked to multidrug resistance.^55^ While it is possible that *blaTEM-1* drives the increase in *E.coli* species observed in our study of rib fractures, distinguishing whether resistance genes regulate the abundance of specific bacteria or if bacteria influence resistance genes remains challenging.

Vancomycin-resistant enterococci^56^ are classed among the serious AMR threats in the United States according to the Centers for Disease Control and Prevention’s 2019 report.^57^ While we did not find an increase in *Enterococci* in the current study, we did observe an increase in the *vanC* gene at 12 weeks post-discharge in the antibiotics compared to the non-antibiotics group. Vancomycin-resistant enterococci acquisition has been linked to antibiotic treatment with flucloxacillin,^58^ which was administered to all antibiotic-treated patients in our study. The vanC gene cluster is known to confer low-level resistance to vancomycin^59^ and therefore is not as epidemiologically significant as the *vanA* and *vanB* high-level resistance-conferring clusters.

The most consistent effect of the antibiotic was the increase of the two *aph* genes, i.e., genes aminoglycoside phosphotransferases which typically confer resistance to aminoglycoside antibiotics such as gentamicin that all the patients in the antibiotic group received.^60^^,^^61^ A number of genes not directly related to the specific antibiotics administered also increased. These included genes encoding resistance to tetracycline (*tetO*, *tetA46*, and *tetQ*),^62^ and a chloramphenicol acetyltransferase enzyme (*catS*) conferring resistance to chloramphenicol.^63^ This could be explained by transferable resistance through plasmids known as transposons or jumping genes.^64^ For instance, transposition of a tetracycline-resistance determinant (Tn1523) is known to be mediated by the pIP231 plasmid in *E.coli*, which is a possible mechanism at play here.^65^ The dysbiotic microbiome may be providing favorable conditions for the exchange of resistance genes between different bacterial species.

The concurrent increase in AMR genes for *bla*, *aph*, *tet*, and *catS* in patients receiving multiple antibiotics underscores the complexity of AMR dynamics. For example, *tet*(O) and *cat* clustered together and were both increased over time in the group treated with antibiotics. Interestingly, these two genes are located within a 40 kb DNA region of a conjugative mobile element in the genome sequence of *Streptococcus suis* strain BM407.^66^ Conjugative mobile elements are segments of DNA that can transfer between bacteria through a process called conjugation, facilitating the spread of resistance genes.^67^ When bacteria acquire such a mobile element, they may gain resistance to several antibiotics at once, making treatment more difficult, which enhances the spread and persistence of multidrug-resistant bacteria, posing a significant challenge to public health. Furthermore, the *tetB(46)* gene was clustered together with the *tetA(46)* gene but had opposite expression patterns in response to antibiotic treatment, with *tetA(46)* being upregulated and *tetB(46)* being downregulated. It has been suggested that both these genes are necessary for tetracycline resistance in *Streptococcus australis* despite encoding distinct tetracycline efflux pump proteins both with the same function of exporting tetracycline out of the cell.^68^ This may suggest a regulatory mechanism that balances the expression of these genes and that they might act sequentially rather than simultaneously.

In addition, the correlation between specific AMR genes and bacterial genera like *Roseburia faecis*, *Enterococcus*, and *Klebsiella* further highlights the complex interplay between antibiotic use, resistance gene prevalence, and bacterial abundance. The genes responsible for AMR are physically associated with mobile genetic elements, allowing them to be horizontally transferred to distantly related microbial species. As a result, the AMR genes present in the chromosomes of commensal gut bacteria could serve as a reservoir of resistance traits for other enteric pathogens. Specifically, *R. faecis* was correlated with the presence of genes conferring resistance to tetracyclines and chloramphenicol, suggesting that the correlation of these AMR genes with *R. faecis* could be a result of horizontal gene transfer. *In vitro*, *R. faecis* produces a bacteriocin-like substance, a type of antimicrobial generated by certain microbes that prevents the growth of related or identical species.^69^ Lactic acid bacteria, which are commonly thought of as probiotics, can also produce this substance.^70^ Consistent with our data it has been previously reported that, similar to pathogenic bacteria, enteric commensals also exhibit multidrug resistance.^71^ *Enterococcus* and *Klebsiella*, known for their role in hospital-acquired infections,^72^ were correlated with aminoglycosides AMRs likely as a result of the exposure to these antibiotics and tetracyclines-resistant genes likely as a result of horizontal gene transfer. This indicates that these genera might harbor and potentially disseminate these resistance genes within the hospital environment or the gut microbiota. Understanding these relationships is essential for developing strategies to combat AMR and ensure effective treatment options remain available.

We note several strengths to the current study. In spite of this being a pilot study, we used in depth state-of-the-art metagenomic characterization to assess changes in AMR which facilitate the unbiased characterization of AMR genes without the need to target specific genes.^73^ The RCT nature of the pilot data means that the two groups are comparable with regards to the type of injury that led to hospital admission. Furthermore, the control group was closely matched to the demographics of the antibiotic group. Lastly, the extended duration of the follow-up allowed us to study changes in genes and bacteria abundances long after the cessation of the antibiotics. This study represents such an analysis conducted within a patient cohort and an intervention setting.

### Limitations of the study

We also acknowledge a number of limitations. Firstly, the limited sample size and pilot nature of the data mean that the conclusions need replication in order to be generalizable. However, we note that the only other study on antibiotic response and AMRs published was conducted on a similar sample size of 10 participants.^50^ Moreover, other longitudinal studies examining the gut microbiome, but not AMRs, in trauma patients have also been fairly limited in sample size given the challenges in collecting prospective samples in this type of clinical setting.^22^^,^^74^ For instance, Howard et al.^22^ followed twelve patients for 72 h, while Hayakawa et al.^74^ followed fifteen patients for up to 14 days. A larger observational study utilizing 16S sequencing and which did not examine AMRs tracked participants for up to 15 days.^75^ However, given the strength of our pilot study as outlined earlier, we believe that it holds potential to inform future study designs. Furthermore, the antibiotic group was individuals who also had surgery, whereas none of those in the group without antibiotics had rib fixation surgery. It is possible that some of the differences observed may be due to surgery and not the antibiotics. For example, in animal models, anesthesia and surgery induce changes in the gut microbiome, particularly a reduction in the abundance of *Lactobacillus*.^76^ In humans, a study aimed to determine the relative contributions of bariatric surgery, perioperative antibiotics, and caloric restriction on intestinal microbiota composition prior to substantial weight loss following surgery. The authors found that perioperative antibiotics, rather than the surgical procedure or caloric restriction itself, drive much of the early changes in the intestinal microbiota observed after bariatric surgery.^77^ Future studies should seek to dissect the effects of anesthesia and surgery on the gut microbiome from the effect of the antibiotics, as no studies on the effects of orthopedic surgery on the gut microbiome in humans exist to date, though animal models have revealed significant dysbiosis in response to antibiotic use.^78^ At any rate, this does not detract from the fact that in this population we see a significant worsening of gut microbiome composition (based on the abundance of *E.coli*, low alpha diversity, and high levels of several AMR genes) even 12 weeks after hospital discharge. We observe a gender imbalance between the two patient groups; however, the differences seen with regards to the no-antibiotics group are the same as those seen versus the age- and sex-matched controls, suggesting that this is unlikely to have introduced a substantial bias. We also observe a difference in sequencing depth between patient groups and controls. To address these limitations, we conducted a sub-analysis using a separate control group of eleven individuals with a ratio of men to women similar to that of the non-antibiotics group and sequenced at the same depth as the patient cohort. We find the same results as with the original control group (Figures S2 and S3), affirming that gender and sequencing depth disparities have not skewed the results. We acknowledge that female hormonal factors, such as estrogen, the use of oral contraceptives, and ovariectomy, are associated with specific microbial species, with females exhibiting a higher degree of microbial diversity.^79^ However, the differences in alpha diversity observed in our study are unlikely to be attributed to gender, as younger adults show a stronger correlation between sex and alpha diversity than middle-aged adults do. In addition, no gender differences in alpha diversity were observed when the participants’ average age was 60 years^80^ like in our case, potentially due to the decline in estrogen levels in menopausal women.^81^ We also used this control group to test for differences in AMR abundances between fracture patients and controls (Figure S4). AMRs, especially those belonging to the lincosamide nucleotidyltransferase gene family, have been found to be more prevalent in females, which was linked to a higher frequency of prescriptions for macrolide-lincosamide-streptogramin antibiotics.^79^ Although we did not find any differences in lincosamide nucleotidyltransferase genes between antibiotics-treated and untreated, it is likely that some differences in AMRs may be due to previously prescribed antibiotics not accounted for by our analysis. We find that AMRs related to the antibiotics received (i.e., *aph(6)-Id_1_M28829*, *aph(3″)-Ib_5_AF321551* and *blaTEM-1A_1_HM749966*) were higher in the antibiotics compared to the controls. Additionally, the presence of the non-antibiotic-related gene *tetA46* was elevated in the antibiotics group, whereas the rest of the AMRs that changed in the antibiotics versus the non-antibiotics group over time showed no significant differences to the controls. This suggests that these genes changed over time in the antibiotics group but are not necessarily uniquely present in the patients receiving antibiotics. Notably, the vancomycin-resistant gene, *VanC2XY_1_EU151754*, was exclusively detected in the antibiotics group due to insufficient coverage in the control group.

### Conclusions

In conclusion, this pilot study of microbiome changes in severe orthopedic trauma patients has shown that gut microbiome composition does not return to a normal state after antibiotic therapy even after 12 weeks. Lower alpha diversity, higher abundance of pathobionts, and increases in acquired AMR genes persist for 12 weeks. Although the pilot data shown here cannot be generalized, these observations have important clinical implications by showing that gut dysbiosis post-antibiotic use after severe trauma is not reversed within 12 weeks. Specifically, the significantly lower alpha diversity in the gut microbiome of these patients is concerning because reduced microbiome diversity is associated with various negative health outcomes, including a weakened immune system, increased susceptibility to infections, and potentially other chronic conditions.

This suggests that probiotic or symbiotic therapies may be an important consideration in order to restore a gut microbiome resilient to the proliferation of pathogens (such as *Enterobacteriaceae*). Potential future research directions stemming from our findings include research into the use of specific probiotics, prebiotics, or synbiotics that can help restore and maintain gut microbiome diversity in orthopedic trauma patients receiving antibiotics. Other options for reducing the increase of pathogens and gut dysbiosis might be to opt for local antibiotic delivery at the surgical site, rather than systemic IV or oral antibiotics. Future studies might therefore focus on developing surgical and perioperative care protocols for more targeted use of antibiotics in surgical patients, such as through local surgical site delivery rather than systemic IV delivery, minimizing unnecessary exposure and reducing the risk of resistance development. In addition, it might promote research for the development of microbiome-sparing antibiotics in humans. For example, a recent study in mice revealed that a new antibiotic lolamicin is selective toward killing pathogenic gram-negative bacteria and does not target commensal bacteria.^82^

## Resource availability

### Lead contact

Further information and requests for resources and reagents should be directed to and will be fulfilled by the lead contact, Afroditi Kouraki (afroditi.kouraki1@nottingham.ac.uk).

### Materials availability

This study did not generate new unique materials/reagents.

### Data and code availability

• The gut microbiome metagenomics data are available on NCBI: PRJNA1110178 (https://www.ncbi.nlm.nih.gov/bioproject/PRJNA1110178). This accession number is also listed in the key resources table. The clinical data used in this study are held by the Department of Academic Orthopedics and will be released to bona fide researchers from the lead contact upon request.
• This paper does not report original code. The analysis was based on code provided within the vignettes of the cited packages, as described in the Quantification and Statistical analysis section.
• Any additional information required to reanalyze the data reported in this paper is available from the lead contact upon request.

## Acknowledgments

This work was supported by the National Institute for Health Research Nottingham Biomedical Research Centre and by 10.13039/501100000272NIHR grants NIHR132240 (OPERA) and 16/61/10 (ORiF) to B.J.O. Support was also provided by UKRI/MRC grants MR/W026813/1 and MR/Y010175/1 to A.M.V. and C.M. C.M. is funded by the 10.13039/100011721Chronic Disease Research Foundation. For the purpose of open access, the authors have applied a CC BY public copyright to any Author Accepted Manuscript version arising from this submission.

## Author contributions

Conceptualization, A.Kouraki, B.J.O., and A.M.V.; methodology, A.Kouraki and A.M.V.; data curation, D.B., A.Kouraki, and A.M.V.; software, A.Kouraki, and A.M.V.; investigation, A.S.Z., A.V., and A.Kelly; formal analysis, A.Kouraki, A.M.V., and D.B.; writing – original draft, A.Kouraki and A.M.V.; writing – review and editing, all authors; funding acquisition, A.M.V. and B.J.O.; resources, J.N., M.B., D.B., A.V., W.A., A.Kelly, A.S.Z., G.T., A.B., and C.M.; supervision, A.M.V.

## Declaration of interests

D.B., A.B., G.T., and M.B. are employees of Prebiomics srl.

## STAR★Methods

### Key resources table

**Table:** 

REAGENT or RESOURCE	SOURCE	IDENTIFIER
**Biological samples**		

Human metagenomics gut microbiome data	Cases (OPERA) and controls	NCBI: PRJNA1110178 (https://www.ncbi.nlm.nih.gov/bioproject/PRJNA1110178)

**Deposited data**		

Human metagenomics gut microbiome data	Cases (OPERA) and controls	NCBI: PRJNA1110178 (https://www.ncbi.nlm.nih.gov/bioproject/PRJNA1110178)

**Software and algorithms**		

TrimGalore (version 0.6.6)	https://zenodo.org/badge/latestdoi/62039322	N/A
BowTie2 (version 2.3.4.3)	https://doi.org/10.1038/nmeth.1923	N/A
MetaPhlAn (version 4.0.6)	https://doi.org/10.1038/s41587-023-01688-w	N/A
HUMAnN (version 3.8)	https://doi.org/10.7554/eLife.65088	N/A
UniRef 90	https://doi.org/10.1093/bioinformatics/btu739	
KMA (version 1.4.11)	https://doi.org/10.1186/s12859-018-2336-6	N/A
ResFinder 7 (version 2022)	https://doi.org/10.1099/mgen.0.000748	N/A
R (version 4.3.2)	https://www.r-project.org/	N/A
‘phyloseq’ R package (version 1.44.0)	https://doi.org/10.1371/journal.pone.0061217	N/A
‘vegan’ R package (version 2.6–4)	http://vegan.r-forge.r-project.org/	N/A
‘DESeq2’ R package (version 1.40.2)	https://doi.org/10.1186/s13059-014-0550-8	N/A
‘DEGreport’ R package (version 1.40.1)	https://doi.org/10.18129/B9.bioc.DEGreport	N/A
GraphPad PRISM 10	https://www.graphpad.com/	N/A

### Experimental model and subject details

#### Study population

##### Patients with multiple rib fractures (OPERA)

Multiple rib fracture patients were recruited from a Level 1 trauma Center in the UK. Inclusion criteria were: adult patients (16 years and above) presenting with multiple (3+) rib fractures suitable for surgical repair (as defined by BOA British Orthopedic Association Standards for Trauma and Orthopedics (BOASts)), and either: clinical flail chest; respiratory difficulty requiring respiratory support or uncontrollable pain using standard modalities. Patients were excluded if: they had a head or thoracic injury requiring emergency intervention; could not be operated on within 72 h. The OPERA cohort was collected as part of The Operative Rib Fixation (ORiF) Study a randomised controlled trial assigning individuals either to standard conservative care or to surgical rib fixation (which requires prophylactic antibiotic use). In total, stool samples were analyzed from 16 individuals who were hospitalised for multiple rib fractures and were collected at the time of hospital discharge (*n* = 9), 6 and 12 weeks post discharge (*n* = 7). Of these *n* = 12 had received prophylactic antibiotics during their hospital stay.

##### Controls

Ten controls of the same average age and sex who had donated stool samples for a separate study were selected and their samples also underwent shotgun sequencing. A different set of eleven controls from a separate study were used for sub-analyses included in the supplementary material.

#### Ethics

The OPERA ORiF (REC ref. 18/SC/066, IRAS 248,460, IRSCTN 10,777,575) and the two control cohorts (REC ref. 18/EM/0154, clinicaltrials.gov registration NCT03545048, and REC ref. 18/WM/0066, clinicaltrials.gov registration NCT03442348) were approved by REC. All participants provided written informed consent.

### Method details

#### Surgical fixation

The surgical stabilization involved immobilizing the fractured structures (ribs, costal cartilages, and sternum), a method increasingly used in the NHS and endorsed by NICE guidance (IPG361). This stabilization was achieved using various metal structures such as plates and screws. The procedure was performed via a thoracotomy, with the stabilizing structure placed either intramedullary or external to the rib.^83^ Specifically, the patient was under general anesthesia. An open approach through an incision was made over the rib fractures to be treated, and the fractured ribs are reduced under direct vision. The affected ribs were stabilized using metal plates or splints, fixed with screws. The fixation may have involved 'rib splints,' which are plate constructs fixed with a screw inserted within the intramedullary canal of the rib. Rib plates or splints were contoured to fit the rib and applied to the outer surface. Any lung and vascular injuries were addressed as necessary during the surgery. The technique for fracture reduction and the number of fractures reduced were left to the discretion of the treating surgeon.

#### Supportive management

All patients, whether randomised to surgery or not, received supportive management. Patients were managed by a consultant-led trauma team in a multidisciplinary, multispecialty manner. The supportive management approach included: (a) resuscitation following the Advanced Trauma Life Support pathway, utilizing new technologies such as trauma CT, tranexamic acid, and thromboelastography, (b) immediate management of associated thoracic injuries (e.g., haemothorax, respiratory compromise, pneumothorax) in the resuscitation room, (c) early contrast CT scanning with 3D surface-rendered images of the thorax for accurate diagnosis, (d) pain management according to a protocol, with access to neuraxial and opioid analgesia as appropriate, (e) insertion of a large bore trauma chest drain using a sterile open technique for patients with moderate haemothorax or pneumothorax, performed by a qualified doctor and (f) ongoing care by the team, including specialist consultant-led surgical, intensive care, pain management, and physiotherapy teams, in a designated trauma ward or intensive care facility.

**Antibiotic use** for the patients was recorded as part of the study. The specific antibiotics and duration are listed in Table S1.

#### Stool sample collection

Rib fracture patients donated stool samples at the time when they were discharged. They were also given a stool collection kit and leaflet with detailed instructions on how to collect and post the sample to the assessment center in the prepaid postbox at 6 weeks and 12 weeks after hospital discharge. The same collection kits were used by all the control individuals. All stool samples were stored at −80°C or at −20°C temporarily until they could be transported to −80°C freezers in the Clinical Science Building at City hospital following local standard operating procedures (SOPs) prior to analysis. SOPs were used at the collection sites and when transferring the samples which were handled by trained research personnel to ensure the high quality and reliability of the research data. Stool samples were then sent to Prebiomics (TN, Italy) for DNA extraction, sequencing quality control, preprocessing and bioinformatics.

#### DNA extraction, library preparation and sequencing

The stored stool samples were processed using the DNeasy 96 PowerSoil Pro QIAcube HT Kit (Qiagen, #47021) for isolating the microbial DNA. The DNA concentration was measured using the Quant-iT 1X dsDNA Assay Kits, BR (Life Technologies, #Q33267) in conjunction with the Varioskan LUX Microplate Reader (Thermo Fisher Scientific, #VL0000D0). At the end of this process, the DNA was diluted in water to the volume indicated by the Illumina protocol, in order to proceed with the subsequent library preparation. The sequencing libraries were generated using the Illumina DNA Prep, (M) Tagmentation (96 Samples, IPB) kit (Illumina, #20060059). The amplified libraries were purified using the double-sided bead purification method, following the Illumina protocol. The DNA concentration (ng/μL) of these libraries was determined using the Quant-iT 1X dsDNA Assay Kits, HS (Life Technologies, #Q33232) and the Varioskan LUX Microplate Reader (Thermo Fisher Scientific, #VL0000D0). Furthermore, the length of the base pair (bp) was determined using the D5000 ScreenTape Assay (Agilent, #5067–5588/9) in conjunction with the TapeStation 4150 (Agilent Technologies, #G2992AA). By considering both the concentration of the library and the length of the base pairs, the appropriate volume of the library to combine in a single tube was determined in order to attain the most favourable cluster density. The library pool was measured using the Qubit 1x dsDNA HS kit (Life Technologies, #Q33231) with the Qubit 3.0 Fluorometer (Life Technologies, #Q33216). The length of the base pairs (bp) was analyzed as described above. The library pools were sequenced using the Novaseq 6000Dx platform (Illumina), with an average depth of 27 Gb per sample for the OPERA patients.

#### Preprocessing and quality control

The sequenced samples underwent preprocessing and quality checking using a standalone pipeline accessible at https://github.com/SegataLab/preprocessing. TrimGalore^84^ (version 0.6.6) was employed to perform read-level quality control. Reads that had a quality score lower than 20, fragmented short reads with a length less than 75, and reads containing more than 5 ambiguous nucleotides were eliminated. For the purpose of identifying contaminants in the DNA screening process, the human DNA (hg19) and Illumina spike-ins PhiX DNA were eliminated from the metagenomic samples using BowTie2^85^ (version 2.3.4.3). The cleaned reads were splitted and sorted.

#### Taxonomic and functional potential profiling

The taxonomic quantification of the microbial communities at the species-level genome bins (SGBs) was performed using MetaPhlAn^32^ software (version 4.0.6). The analyses were conducted using the default parameters and the most recent version of the MetaPhlAn database (mpa_vOct22).^86^ The software HUMAnN^87^ (version 3.8) was used to investigate the functional capabilities of the microbial communities. This analysis was conducted at the gene family level, utilising UniRef90 gene families, and at the pathway level, using the MetaCyc database.^88^

#### QC for taxonomic annotation

The MetaPhlAn database of sequence-defined species-level genome bins (SGB)-specific markers, started with a catalog of 729,195 dereplicated and quality-controlled genomes (560,084 MAGs and 169,111 reference genomes) leading to the definition of 21,373 FGBs, 47,643 genus-level genome bins (GGBs) and 70,927 SGBs, 23,737 of them containing at least one reference genome (known SGBs) and 47,190 containing only metagenome assembled genomes (MAGs) (unknown SGBs). To minimize the chance that SGBs incorporate assembly artifacts or chimeric sequences, uSGBs with at least five MAGs (no filtering for known SGBs) were used. The genome catalog was then annotated using the UniRef90 database^89^ and, within each SGB, the genes that could not be assigned to UniRef90 gene families were *de novo* clustered together using the UniClust90^90^ criteria (>90% identity and >80% coverage of the cluster centroid). Using the resulting UniRef- and UniClust90 annotations, a set of core genes was defined for each quality-controlled SGB (genes present in almost all genomes composing an SGB), and after mapping all core genes against the entire genomic catalog, we defined a set of 5.1 M SGB-specific marker genes (core genes not present in any other SGB) for a total of 21,978 known SGBs and 4,992 unknown SGBs. For the taxonomic profiling step that uses the markers based on the SGB data, MetaPhlAn 4 maps metagenomic reads against the marker database using Bowtie 2.^85^

#### Antimicrobial resistance genes profiling

Metagenomics makes it possible to sequence all the DNA from a sample. Then with the help of alignment based tools, antimicrobial resistance (AMR) genes can be identified based on their sequence identity with known AMRs. KMA uses k-mer seeding to increase mapping speed and is specifically made for mapping reads against redundant databases. KMA was developed with the task of aligning raw sequence data directly to redundant databases such as ResFinder (http://genepi.food.dtu.dk/resfinder). ResFinder identifies acquired genes and/or finds chromosomal mutations mediating antimicrobial resistance in total or partial DNA sequence of bacteria. Species-specific predictions for selected antimicrobial agents are also implemented in ResFinder in order to only present clinically relevant phenotypes for these species and also to include mutation-mediated phenotypes. Currently these include *Campylobacter coli*, *Campylobacter jejuni*, *Enterococcus faecalis*, *Enterococcus faecium*, *Escherichia coli*, *Mycobacterium tuberculosis*, *Salmonella* and *Staphylococcus aureus*.^91^ The software KMA^92^ version 1.4.11, was utilised to evaluate the prevalence of AMR genes in the microbial communities. The preprocessed reads were aligned against the ResFinder 7 database (version 2022) to assign antimicrobial resistance genes to all our microbiomes. The benchmarking that was done for the publication of ResFinder 4.0 showed concordance between the genotypical predicted and phenotypically detected phenotypes above 95% on overall average. Only hits having sufficient alignment coverage and identity (at least 90%) and a satisfactory depth of coverage of the template (at least 5-fold) were taken into account. The abundance of AMR-related genes was further standardised using the reads per kilobase million (RPKMs) metric.

### Quantification and statistical analysis

Statistical analyses were performed using GraphPad Prism (version 10.2.0) and R (version 4.3.2).

#### Alpha diversity

The Shannon diversity index also called Shannon Entropy^93^ was calculated based on the species-level data and using the estimate_richness function from the ‘phyloseq’ package (version 1.44.0).^94^ Mann-Whitney U Tests were used to compare the controls and the antibiotics groups at the different timepoints.

#### Beta diversity

To quantify the dissimilarity between the antibiotic samples and controls from all timepoints and between the antibiotic samples and those from patients who had not received prophylactic antibiotics at baseline we computed the Bray-Curtis dissimilarity matrix at the species-level using the ‘vegdist’ command from the ‘vegan’ package (version 2.6–4). Bray-Curtis takes into account both the presence and abundance of different species within the samples.^95^ All distances were extracted from the same distance matrices and were displayed in violin plots. Statistics on the distances were performed using Mann-Whitney U Test and Permutational multivariate analysis of variance (PERMANOVA) adjusted for time as implemented in the adonis2 function of the ‘vegan’ package (version 2.6.4).^96^ PERMANOVA is a statistical method used in ecology and bioinformatics to analyze patterns of dissimilarity among groups of samples, such as those obtained from metagenomic data and operates by testing the significance of differences in multivariate dispersion between groups of samples assessing whether the multivariate means or “centroids” of groups of samples are different, whilst taking into account the dispersion around the centroids.

#### Enterobacteriaceae

Since *Escherichia coli* was the most abundant species in the surgical cohort, we then examined changes in both *Escherichia coli* and related taxonomic groups, including *Gammaproteobacteria* (its class) and *Enterobacteriaceae* (its family). Raw abundances were transformed using the additive log-ratio transformation to account for the compositional nature of the data.^97^ Mann-Whitney U Tests were employed on the transformed data to compare the controls and the antibiotics groups at the various timepoints. There were no zero counts for the class *Gammaproteobacteria*, the family *Enterobacteriaceae* and the species *Escherichia coli* in surgical patients, with these being the most abundant taxa. In the controls group, one participant had a zero count, which was replaced with a small pseudo-count following previously established methods for handling zero counts in microbiome data.^98^

#### AMR genes

The ‘DESeq2’ (Differential Expression analysis for Sequencing data) package (version 1.40.2) was used to investigate changes in the abundance of AMR genes following treatment with antibiotics across the various timepoints.^99^^,^^100^ DESeq is a method used to analyze RNA-Seq data, particularly for comparing gene expression levels between different conditions or treatments. DESeq is widely used in bioinformatics and genomics for differential analysis of count data aimed at giving better estimate interpretability and stability. This analysis controls for differences between groups at baseline, including random differences between the individuals from each group, which are observable before the antibiotic takes effect. An interactive model was used that included the interaction term (timepoint:antibiotic) to identify all the AMR genes that show a significant and consistent change (both nominally and at false discovery rate) in abundance between the antibiotics and non-antibiotics groups from baseline onward. The Wald test was used to conduct individual comparisons between antibiotic-treated and non-treated from baseline to follow-up (at 6 and 12 weeks). The data were normalised using the "poscounts" estimator that deals with genes with some zeros, by calculating a modified geometric mean by taking the n-th root of the product of the non-zero counts specifically evolved for use with metagenomic samples. Here is a detailed explanation of how we applied DESeq to our time series data.

(1) Design Formula: We created a design formula to model three key aspects:
(2) Difference at Time 0: Baseline differences between groups
(3) Difference Over Time: Changes within each group over the study period.
(4) Group-Specific Differences Over Time (Interaction Term): Represented as ‘timepoint:antibiotic’, this term captures the interaction between time and group (e.g., antibiotic vs. non-antibiotic). It accounts for how the changes over time differ between the groups after adjusting for baseline differences.
(5) Interaction Term: This is crucial as it identifies the difference between the two groups at any given time point, considering the baseline differences. For example, it helps to see how the antibiotic group’s response over time differs from the non-antibiotic group, after considering their initial states at time 0.
(6) Likelihood Ratio Test: This test was used to evaluate the significance of the group-specific differences over time by removing these differences and identifying if the interaction term (group-specific changes over time) adds meaningful information.
(7) Wald Tests for Log2 Fold Changes: For each individual time point, we used Wald tests to examine the log2 fold changes. This involves using the ‘test’ argument in DESeq’s ‘results’ function to specifically assess how much each AMR gene’s expression changes at each time point relative to time 0. AMRs with small p-values from the Wald tests indicate significant group-specific effects at particular time points after time 0. This means that these AMRs show notable differences between the antibiotic and non-antibiotic groups at specific times, which are not attributable to random baseline differences.
(8) Controlling for Baseline Differences: By including the initial time point and interaction terms in the design, DESeq controls for pre-existing differences between groups. This ensures that any observed changes in AMRs are due to the intervention (e.g., antibiotic administration) rather than inherent differences between the individuals in each group.

In summary, the DESeq method allowed us to effectively model and analyze the complex interactions and changes over time in our study, providing insights into how specific AMRs respond to antibiotics while controlling for baseline differences.

#### Associations between AMR genes and bacteria

Correlations between bacterial taxa and AMRs that were significantly different between the antibiotics and non-antibiotics groups were explored using Spearman’s correlation. The abundance data was first rarefied, retaining only species level with abundances >0.1% and present in at least 25% of all samples in the study. This left a total of 266 species. Genus level was also considered if no species in the genus achieved the abundance and prevalence of the cut-off but the genus did, this was the case for 19 genera making the total number of taxa tested equal to 285. *p*-values <0.05 from the Spearman’s rho tests were considered nominally statistically significant and were denoted with a single asterisk, while correlations with *p* < 0.1 after false discovery correction were considered FDR statistically significant and were marked with three asterisks. Comparisons of the percentage contribution of the relative abundance of selected pathogenic and short-chain fatty acid producing species between AMR negative and AMR positive individuals of selected significantly differentiating AMRs were performed using Mann Whitney U tests. Asterisks for statistical significance are displayed as: ∗∗*p* < 0.01, ∗∗∗*p* < 0.001, ∗∗∗∗*p* < 0.0001.

#### AMR gene clusters

To identify AMR genes associated with particular patterns across sample groups we used the clustering tool degPatterns from the ‘DEGreport’ package,^101^ which groups the genes based on their changes in expression across sample groups. This clustering tool uses a hierarchical clustering approach based on pairwise correlations, then cuts the hierarchical tree to generate groups of genes with similar expression profiles. The tool cuts the tree in a way to optimise the diversity of the clusters, such that the inter-cluster variability is larger than the intra-cluster variability. We only analyzed the AMR genes that significantly increased from baseline to follow-up (at 6 and 12 weeks) and changed between the antibiotics compared to the non-antibiotic groups as identified from the DESeq analyses above as this method does not calculate significant differences between groups. However, *tetB(46)*, which was significantly decreased in DESeq, was included in the analysis because it, along with *tetA(46),* is suggested to be necessary for tetracycline resistance in *Streptococcus australis*, despite encoding distinct tetracycline efflux pump proteins that both function to export tetracycline out of the cell.^68^ We used this clustering approach to identify clusters based on overall expression patterns across multiple timepoints of the AMRs of interest between the antibiotic and the non-antibiotic groups and identify groups of AMRs that group together based on their changes in expression across sample groups. The 'regularised log' transformed AMR data were used as input for this analysis. The normalised values (*Z* score of gene abundance) from the clustering were extracted to create the bar plots.

#### Link between the shift in microbiome diversity, composition, and AMRs with health endpoints

We also investigated whether there a relationship between the shift in microbiome diversity, composition, and AMRs with demographics and health endpoints (Figure S1) using Spearman’s rho correlations between alpha diversity (Shannon index), relative abundance (additive log transformed) of *E.coli*, and abundance of antimicrobial resistance genes with age, sex, length of hospital stay (LOS), days spend at the intensive care unit (ICU), quality of life measured with EuroQol- 5 Dimension questionnaire (EQ5d) and pain (measure with a visual analogue scale; VAS) at the 3-month (90-day) follow-up. The EQ5d includes one question for each of the five dimensions that include mobility, self-care, usual activities, pain/discomfort, and anxiety/depression.^38^ Asterisks for statistical significance are displayed as: ∗*p* < 0.05 and ∗∗*p* < 0.01.

#### Sub-analysis

Due to a gender imbalance between the two patient groups and a difference in sequencing depth between patient groups and controls, we performed a sub-analysis using a separate control group of eleven individuals. This group was matched to the non-antibiotics group in terms of age (average 61.36 years, SD 10.22) and sex (73% male) and was sequenced at the same depth as the patient cohort. We conducted Mann-Whitney U tests to assess differences in Shannon (alpha diversity) and Bray-Curtis (beta diversity) indices (Figure S2), as well as the mean abundance (additive log transformed) of the class *Gammaproteobacteria*, family *Enterobacteriaceae*, species *Escherichia coli*, and the mean abundance of AMRs, including *tet(A)_6_AF534183*, *aph(6)-Id_1_M28829*, *aph(3″)-Ib_5_AF321551*, and *blaTEM-1A_1_HM749966* (Figures S3 and S4). *VanC2XY_1_EU151754* was not detected in the control group due to insufficient coverage, and other AMRs that changed significantly over time in the antibiotics group compared to the non-antibiotics group did not show significant differences between cases and controls. The comparison between fracture cases (with and without antibiotic use at baseline and/or 12 weeks) and the matched control group revealed similar results to those obtained with the original control group as discussed in the “Limitations of study” section.
