# Supplementary material for: Metagenomic changes in response to antibiotic treatment in severe orthopedic trauma patients
Source: iScience. 2024 Aug 22;27(9):110783. doi: 10.1016/j.isci.2024.110783 (PMC11403444; doi:10.1016/j.isci.2024.110783)
Supplement: Document S1. Figures S1–S4 and Table S1 [file mmc1.pdf]

iScience, Volume *27*

Supplemental information

Metagenomic changes in response to antibiotic treatment in severe orthopedic trauma patients

Afroditi Kouraki, Amy S. Zheng, Suzanne Miller, Anthony Kelly, Waheed Ashraf, Davide Bazzani, Angela Bonadiman, Guendalina Tonidandel, Mattia Bolzan, Amrita Vijay, Jessica Nightingale, Cristina Menni, Benjamin J. Ollivere, and Ana M. Valdes

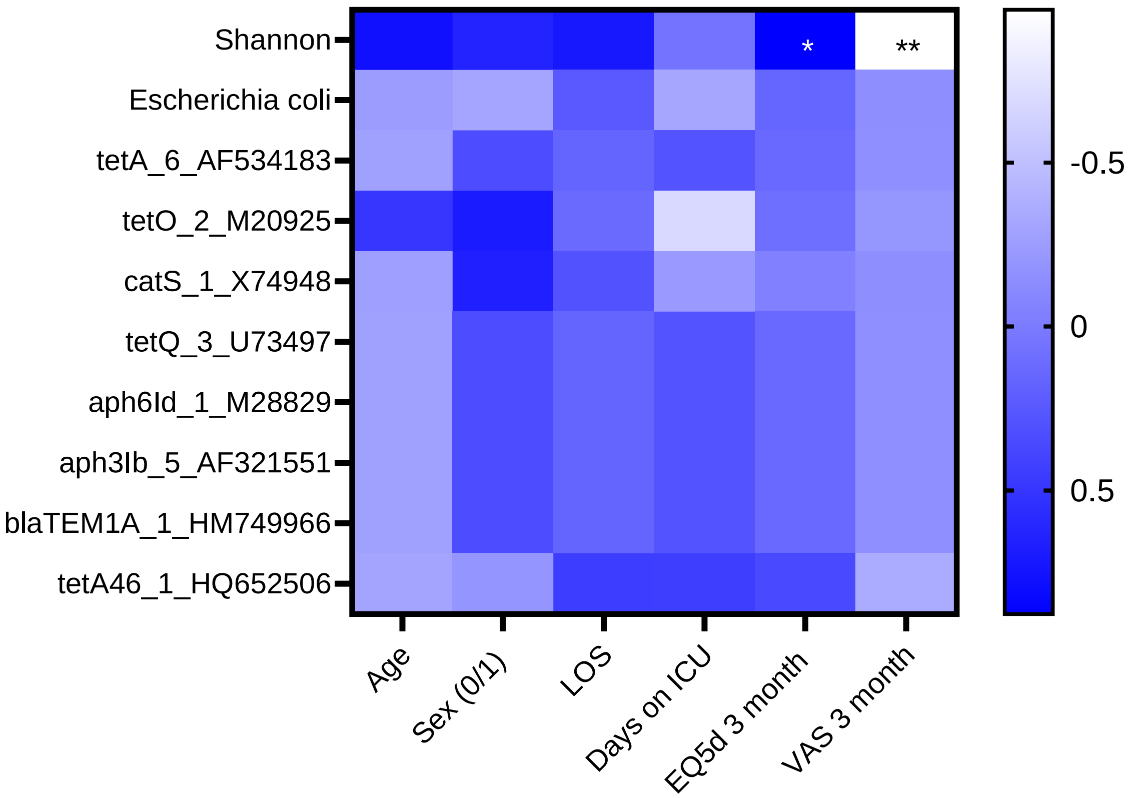

Figure S1. Heatmap plot illustrating correlations between the shift in microbiome diversity, composition, and AMRs with demographics and health endpoints, Related to Star Methods. LOS; length of hospital stay, ICU; intensive care unit, EQ5d; EuroQol- 5 Dimension questionnaire; VAS; visual analogue scale. *$p<0.05$ and **$p<0.01$. p values from Spearman's rho tests. Scale bar ranges from -0.96 to 0.87.

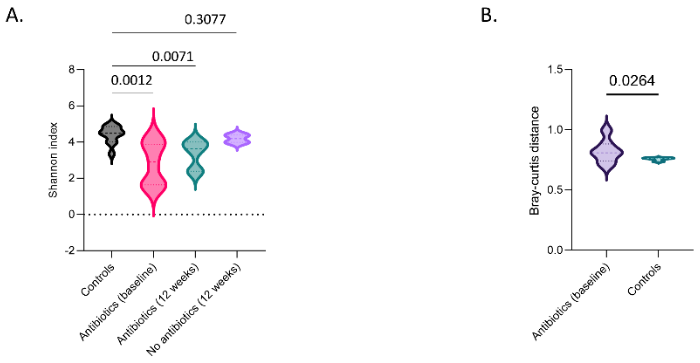

Figure S2. Violin plots of: (A) Shannon (alpha diversity) and (B) Bray-curtis (beta diversity) showing differences between fracture cases and controls, Related to Star Methods. p-values from Mann Whitney U tests

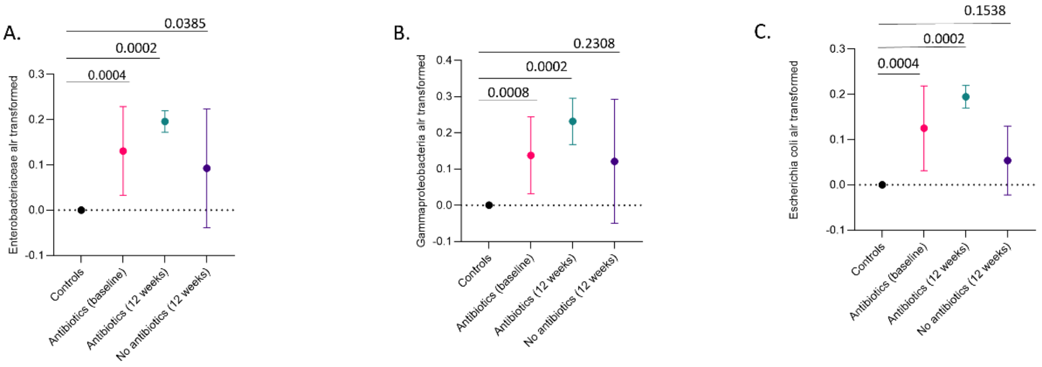

Figure S3. Mean abundance plots of: (A) class *Gammaproteobacteria*, (B) family *Enterobacteriaceae* and (C) species *Escherichia coli* showing differences between fracture cases and controls, Related to Star Methods. alr; additive log transformed. p-values from Mann Whitney U tests

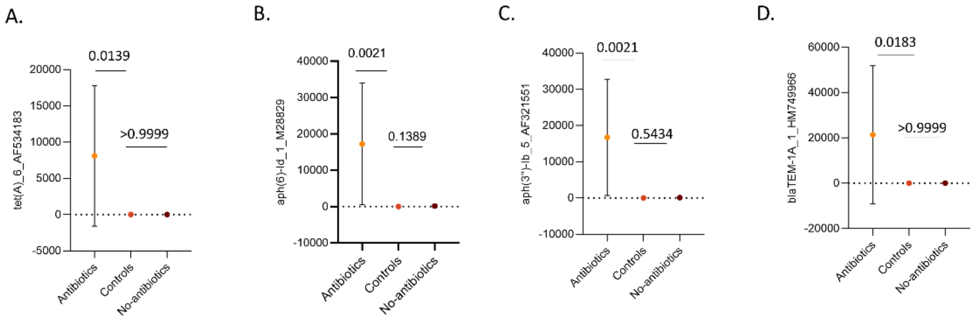

Figure S4. Mean abundance plots of AMRs: (A) *tet(A)_6_AF534183*, (B) *aph(6)-Id_1_M28829*, (C) *aph(3'')-Ib_5_AF321551* and (D) *blaTEM-1A_1_HM749966* between fracture cases and controls, Related to Star Methods. p-values from Mann Whitney U tests.

Table S1. Descriptive characteristics of surgical patients admitted to hospital for multiple rib fractures who received antibiotics or not and controls age and sex matched to the antibiotic group, Related to Figure 1 and Star Methods.

	n	sex M%	age (yrs)	rib fixation surgery %	Antibiotics [(1)] %			
			Mean (SD)		*Flucloxacill in 24hrs*	*Gentamicin 1 dose*	*Tazocin 1dose*	*Co-amoxiclav 5doses*
Controls	10	50.0	63.83 (*9.28*)	0.0%	0.0%	0.0%	0.0%	0.0%
Fracture patients antibiotics	11	55.5	64.35 (10.18)	63.6%	100.0%	90.9%	27.3%	9.1%
Fracture patients no antibiotics	5	80.0	57.60 (*13.05*)	0.0%	0.0%	0.0%	0.0%	0.0%

(1) Flucloxacillin is a narrow-spectrum beta-lactam antibiotic belonging to the penicillin class. It is commonly used to treat infections caused by susceptible Gram-positive bacteria, including *Staphylococcus aureus.* Gentamicin is an aminoglycoside antibiotic that acts by inhibiting protein synthesis in susceptible bacteria. It is effective against a wide range of Gram-negative bacteria and some Gram-positive bacteria. Tazocin, also known as piperacillin/tazobactam, is a combination antibiotic containing piperacillin, a broad-spectrum penicillin, and tazobactam, a beta-lactamase inhibitor. It is used to treat a variety of infections caused by susceptible bacteria, including both Gram-positive and Gram-negative organisms. Co-amoxiclav, also known as amoxicillin/clavulanic acid, is a combination antibiotic consisting of amoxicillin, a penicillin-like antibiotic, and clavulanic acid, a beta-lactamase inhibitor. It is effective against a wide range of bacterial infections, including those caused by beta-lactamase-producing bacteria, and is commonly used for respiratory tract infections, urinary tract infections, and skin infections.
